# *Pneumocystis jiroveci* pneumonia and colonization in patients with advanced lung cancer

**DOI:** 10.3892/ol.2012.1052

**Published:** 2012-11-29

**Authors:** YOSUKE TOGASHI, KATSUHIRO MASAGO, YUTAKA ITO, YUICHI SAKAMORI, CHIYUKI OKUDA, AKIKO FUKUHARA, HIROKI NAGAI, YOUNG HAK KIM, MICHIAKI MISHIMA

**Affiliations:** Department of Respiratory Medicine, Graduate School of Medicine, Kyoto University, Sakyo-ku, Kyoto, Japan

**Keywords:** *Pneumocystis jiroveci* pneumonia, colonization, lung cancer, immunocompromised host

## Abstract

*Pneumocystis jiroveci* pneumonia (PCP) has long been recognized as a cause of mortality in immuno-compromised populations, including those with advanced lung cancer. Although *Pneumocystis* colonization has only recently been described due to the development of more sensitive molecular techniques, including polymerase chain reaction (PCR), it is unknown whether *Pneumocystis* colonization leads to the development of PCP. In the present study, we aimed to determine the prevalence of *Pneumocystis* colonization in advanced lung cancer patients. Furthermore, the association between PCP and *Pneumocystis* colonization was also investigated. Advanced lung cancer patients with no indication of PCP were evaluated to determine the prevalence of *Pneumocystis* colonization. We analyzed their oral wash (OW) samples and retrospectively evaluated advanced lung cancer patients with PCP by analyzing their sections of formalin-fixed, paraffin-embedded lung tissues obtained following a diagnosis of lung cancer. *Pneumocystis* colonization was determined by a PCR test for *Pneumocystis jiroveci (P. jiroveci)*. No *P. jiroveci* was detected by PCR in the OW samples of 47 advanced lung cancer patients with no indication of PCP, or in the lung tissues of four advanced lung cancer patients with PCP. These results indicate that PCP is not associated with *Pneumocystis* colonization in advanced lung cancer patients, although this study is limited since this was a cross-sectional and retrospective study.

## Introduction

*Pneumocystis jiroveci* pneumonia (PCP) is a potentially life-threatening infection that occurs in immunocompromised populations (1). HIV-infected patients with a low CD4 count are at high risk of PCP. Other individuals at substantial risk include hematopoietic stem cell and solid organ transplant recipients, those with cancer and those receiving corticosteroids, cytotoxic agents and other immunosuppressive medications (2–5).

The detection of *Pneumocystis jiroveci (P. jiroveci)* in individuals with no indication of PCP has been defined as colonization. Although *Pneumocystis* colonization was recently described using sensitive molecular techniques, including polymerase chain reaction (PCR), the clinical significance of *Pneumocystis* colonization is not yet fully understood. It is unknown whether *Pneumocystis* colonization leads to the development of PCP (6). In this study, we determined the prevalence of *Pneumocystis* colonization in advanced lung cancer patients in order to investigate the association between PCP and *Pneumocystis* colonization.

## Patients and methods

### Patients

A total of 47 advanced lung cancer patients with no indication of PCP from Kyoto University Hospital (Kyoto, Japan) were evaluated between June 2009 and October 2009 to determine the prevalence of *Pneumocystis* colonization. We also retrospectively evaluated four advanced lung cancer patients with PCP during the period between August 2002 and October 2009 at Kyoto University Hospital. No patient was known to be HIV-positive. Informed consent was obtained from all patients prior to the study.

### Samples

The investigation of patients with no indication of PCP was performed using oral wash (OW) samples from the 47 patients. OW samples were obtained by rinsing and gargling oral cavities with 20 ml of sterile saline for 30 sec. The investigation of patients with PCP was performed using sections of formalin-fixed, paraffin-embedded lung tissues obtained following a diagnosis of lung cancer. Of the five patients, four had no indication of PCP at the time of diagnosis. The sample from the one patient who not analyzed further.

### Sample analysis

*Pneumocystis* colonization was determined by a PCR test for *P. jiroveci*. Formalin-fixed paraffin-embedded tissue was cut into 5-*μ*m sections and mounted onto pre-treated glass slides. The slides were de-paraffinized and DNA was extracted with phenol-chloroform and ethanol precipitation. The PCR analysis for *P. jiroveci* was performed in 50 *μ*l of amplification reaction mixture with denaturation at 943°C for 90 sec, annealing at 503°C for 90 sec and extension at 723°C for 120 sec (40 cycles). The oligonucleotide primers used at 100 pmol were: 5′-GATGGCTGTTTCCAAGCCCA-3′ and 5′-GTGTAGGTTGCAAAGTACTC-3′. DNA products of 376 bp were amplified from template DNAs. This analysis was performed at SRL Inc. (Tachikawa, Tokyo, Japan). The details of this method and its use in the detection of *P. jiroveci* were described by Wakefield *et al*(7).

## Results

### Patients with no indication of PCP

The clinical characteristics of the 47 patients with no indication of PCP are listed in Table I. Although corticosteroids or cytotoxic agents were administered to certain patients, no *P. jiroveci* was detected by PCR in the OW samples.

### Patients with an indication of PCP

The clinical characteristics of the four patients with PCP at the time of diagnosis of lung cancer are listed in Table II. Neither corticosteroid nor other immunosuppressive medication were administered to the four patients upon diagnosis of lung cancer and no *P. jiroveci* was detected by PCR in the lung tissues of the patients. At the diagnosis of PCP, corticosteroids were administered to all four patients. Of the four patients, three received cytotoxic agents following a diagnosis of lung cancer with PCP.

## Discussion

In this study, no *P. jiroveci* was detected by PCR in OW samples of the 47 patients with no indication of PCP. In addition, no *P. jiroveci* was detected by PCR in the lung tissues of the four patients with PCP. These results indicate that PCP was not associated with *Pneumocystis* colonization.

The most significant risk factors for PCP in patients without HIV infection are corticosteroid use and defects in cell-mediated immunity (5,8–10). Cancer is also one of the significant risk factors for PCP and cancer patients often receive cytotoxic agents or corticosteroids (11). In a retrospective study of 80 PCP cases in 79 cancer patients, PCP occurred more often in patients with hematological malignancies (66%), the majority of whom had either leukemia or lymphoma. In addition, 25 patients had solid tumors and 23 patients had undergone hematopoietic stem cell transplantation (11). There were no data with regard to what type of solid cancer tumors were complicated with PCP in this study. In this retrospective study, 5 of 505 patients (1.0%) with advanced lung cancer had complications with PCP. Therefore, PCP is considered to be a rare complication in patients with advanced lung cancer.

The primary mode of transmission of *P. jiroveci* is uncertain. Primary exposure to *P. jiroveci* is common in young children, as is demonstrated by the increase in anti-*Pneumocystis* antibody titers during the first few years of life (7,12). These primary infections were presumed to be asymptomatic and the organism was believed to remain in a latent state unless the patient became immunosuppressed. However, this theory of latency has come under scrutiny. Experiments in rats or mice with severe combined immunodeficiency and macaques infected with simian immunodeficiency virus all show that these hosts are capable of clearing the organism (13–15). *Pneumocystis* colonization is different from this latent infection in that *P. jiroveci* is detected directly. The prevalence of *Pneumocystis* colonization among healthy adults without immunosuppressive conditions or lung disease in previous studies has ranged from 0 to 20% (6). Certain risk factors, including low CD4 cell count, corticosteroid, smoking and COPD appear to affect the risk of *Pneumocystis* colonization (6). Of the 47 patients with advanced lung cancer included in our study, no *Pneumocystis* colonization was detected although there were some patients with these risk factors. Maskell *et al* detected six *Pneumocystis* colonizations in bronchoalveolar lavage from 35 patients with lung cancer (16). The differences in these studies may be explained by varying geographic exposure in the populations, differences in experimental techniques or differing subject characteristics.

Certain diseases, including SIDS and COPD, are associated with a high prevalence of *Pneumocystis* colonization, but the association between *Pneumocystis* colonization and development of these diseases is unclear (6). In our retrospective study, no *P. jiroveci* was detected by PCR in the past lung tissues of the four advanced lung cancer patients with PCP, which indicated that PCP was not associated with *Pneumocystis* colonization. However, this study is limited as it is a retrospective study. To fully assess the association, a prospective study is needed.

## Figures and Tables

**Table I.** Clinical characteristics of the patients without *Pneumocystis jiroveci* pneumonia.

Characteristics	No. of patients
Median age (years)	47
Gender (%)	
Male	36 (77)
Female	11 (23)
Performance status (%)	
0	19 (40)
1	15 (32)
2	12 (26)
3	1 (2)
Smoking status (%)	
Never	13 (28)
Former	34 (72)
Comorbid lung disease	
Chronic obstructive pulmonary disease	9
Interstitial pneumonia	4
Others	6 (asthma 3, radiation pneumonitis 1, bronchiectasis 1, silicosis 1)
Histology (%)	
Non-small cell lung cancer	30 (64)
Small cell lung cancer	17 (36)
Stage (%)	
IV	27 (57)
IIIB	18 (38)
rIV	2 (5)
Treatment (%)	
None	29 (62)
Cytotoxic agents	16 (34)
Others	2 (4) (Gefitinib 1, Stereotactic radiation 1)
Corticosteroid (%)	
Oral	4 (9)
Inhalation	3 (6)
Peripheral blood lymphocyte count (%)	
<1500/*μ*l	24 (51)
≥1500/*μ*l	23 (49)

Former, former-smoker; R, recurrent.

**Table II.** Clinical characteristics of the patients with *Pneumocystis jiroveci* pneumonia upon the diagnosis of lung cancer.

	Case 1	Case 2	Case 3	Case 4
Age (years)	78	47	75	66
Gender	Male	Male	Male	Male
Performance status	1	0	0	0
Smoking status	Former	Former	Former	Former
Comorbid lung disease	COPD	None	COPD	COPD, IP
Histology	Sq	Ad	Ad	Sq
Stage	IIIB	IV	IV	IIIA
Immunosuppression	No	No	No	No
Corticosteroid	No	No	No	No
Lymphocyte (cells/*μ*l)	1800	1600	2600	3900

Former, former-smoker; COPD, chronic obstructive pulmonary disease; IP, interstitial pneumonia; Sq, squamous cell carcinoma; Ad, adenocarcinoma; immunosuppression, immunosuppressive agent; lymphocyte, peripheral blood lymphocyte count.
